# Analysis of the Weighted Kappa and Its Maximum with Markov Moves

**DOI:** 10.1007/s11336-022-09844-y

**Published:** 2022-02-03

**Authors:** Fabio Rapallo

**Affiliations:** Department of Economics, University of Genova, Via Francesco Vivaldi 5, 16126 Genoa, Italy

**Keywords:** algebraic statistics, Markov bases, ordinal data, rater agreement, pairwise agreement, simulated annealing

## Abstract

In this paper, the notion of Markov move from algebraic statistics is used to analyze the weighted kappa indices in rater agreement problems. In particular, the problem of the maximum kappa and its dependence on the choice of the weighting schemes are discussed. The Markov moves are also used in a simulated annealing algorithm to actually find the configuration of maximum agreement.

The analysis of rater agreement is currently one among the most active and relevant research areas in categorical data analysis. Even in the simplest case where two or more observers rate a common set of *n* objects on the same rating scale, there are in literature several indices to summarize the agreement, each of them with its own paradoxes, counterexamples, and unexpected behaviors. Indeed, the large spectrum of possible indices is the symptom of the difficulties in the interpretation of the results. For a general survey, the reader can refer to Fleiss et al. (2003), von Eye and Mun (2004), or Shoukri (2010).

The most popular measures of agreement, at least in the two-rater case, are the Cohen’s $\kappa$ and the weighted Cohen’s $\kappa _w$. First introduced in Cohen (1960) and Cohen (1968), respectively, such two indices have been analyzed, criticized, generalized, in order to adapt to the multi-rater case, to incomplete rating schemes, and so on. For instance, in the multi-rater case the most popular extension of the Cohen’s $\kappa$ is the Conger’s $\kappa _C$ introduced in Conger (1980), where the pairwise agreement in all possible two-way marginal tables is considered, see the discussion and the examples in Vanbelle (2019). In all cases, the rationale behind such indices is the measurement of the rater agreement beyond chance, in the sense that under complete independence of the raters the value of the indices should be zero. In this paper, we restrict our attention to the weighted Cohen’s $\kappa _w$ and its extensions to the multi-rater case, paying special attention to the connections between the choice of the weighting scheme and the maximum attainable value of such indices.

A first issue of the kappa-type statistics we consider in this paper is normalization. It is known that the interpretation of kappa-type statistics is not straightforward since their maximum is 1 only when the marginal distributions are homogeneous. In the case of non-homogeneous margins, the maximum value can be considerably less than 1. As customary in statistics when working with indices, a problem is therefore to compute the maximum value attainable by an index in order to compare the observed value with the maximum. Some attempts has been made in the direction of finding the maximum value of the kappa statistics. For instance, a procedure has been introduced in Umesh et al. (1989), where the maximum agreement is found by fixing the observed agreement and by varying the marginal distributions. When working with fixed margins, however, the computation of the maximum attainable kappa is relatively easy only in the case of the unweighted $\kappa$ in two-rater setting, see, e.g., Sim and Wright (2005). The problem is less simple in the weighted case or in the multi-rater setting. We will illustrate this point extensively.

A second issue this paper deals with is the dependence of the kappa-type statistics on the choice of the weights. The unweighted version of the Cohen’s $\kappa$ only distinguishes between agreement cells and disagreement cells, and thus, it is used in case of ratings on a nominal scale. When the rating scale is ordinal, or in general when there are some disagreements to be considered more serious than others, then the weighted $\kappa _w$ should be preferred. In this case, the choice of the weights is a delicate issue, and it is known that different weighting schemes lead to quite different results. In addition, the main weighting schemes (i.e., linear or quadratic) have been studied extensively, but there is a need of further analyses to better understand the role played by the weights in the behavior of kappa-type statistics. In a recent paper by Kvålseth Kvålseth (2018), the dependence of the weighted $\kappa _w$ on the choice of the weights is highlighted, and the relevance of the interpretation of the $\kappa _w$ values as functions of the weighting schemes is discussed extensively. The author motivates its study on the properties of the weights claiming that, without a clear understanding of the connections between the weights and the $\kappa _w$, the weighted $\kappa _w$ itself is not a satisfactory index to describe the agreement in an ordinal context. Thus, also this problem will be considered here.

Both the points described above are analyzed in this paper with the aid of algebraic statistics. We give insights and new results to provide a precise understanding of the action of weights in the computation of the kappa, and this will help for a correct interpretation of weighted kappa statistics. This analysis is carried out here by means of the Markov moves, well-known tools in algebraic statistics for the analysis of contingency tables. In particular, we show how the properties of the weights affect the configuration of maximum agreement. Our results allow a precise understanding of the role of the weights and their impact on the structure of the configuration with maximum agreement. The use of algebraic statistics for rater agreement analysis has been considered in other works, but mainly for computational purposes. For instance, the use of Markov bases to make exact tests in this framework can be found in Rapallo (2003) and Rapallo (2005).

Without introducing here formal definitions, for which the reader can refer to the next section, let us present a simple example, taken from von Eye and Mun (2004), page 74. Two psychiatrists $P_1$ and $P_2$ rate the severity of depression of 129 patients using a three-level ordinal scale. The observed data are in Fig. 1 (left). A table with the same margins and maximum agreement with linear weights is in Fig. 1 (center), while the table with the same margins and maximum agreement with quadratic weights is in Fig. 1 (right).

**Fig. 1:**
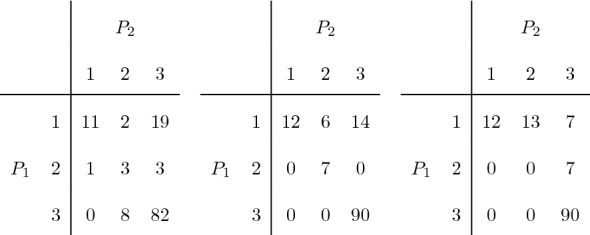
Two psychiatrists’ rating of severity of depression. The observed table (left), a table with the same margins and maximum agreement with linear weights (center), and the table with the same margins and maximum agreement with quadratic weights (right).

Already from this simple example, we can highlight some counterintuitive facts. For instance, not always the maximum agreement is obtained by maximizing the counts on the diagonal. With quadratic weights the maximum is not attained in a table which fulfills the main diagonal as much as possible. We will show that to fulfill the diagonal is a good strategy to increase the agreement only if the weights define a distance on the ground set, and this is not the case, for instance, when quadratic weights are used. On that point, the available resources to compute the maximum agreement fail. The R package rel, LoMartire (2020) and, for the two-rater setting, some online calculators, e.g., in Lowry (2020) at the date of submission, do not give the correct answer.

Another interesting point concerning the above example is that with linear weights there are several configurations with the same value of kappa. The tables in Fig. 1 (center and right) share the same value of weighted kappa with linear weights (0.6089), although they appear rather different at a first sight, and the weighted kappa with quadratic weights ranges from 0.6007 to 0.6909. We will see that in this simple example all tables with the maximum agreement under linear weights can be obtained in an easy way, as only one Markov move can be applied.

Finally, we exploit again the Markov moves, and we introduce a simple simulated annealing algorithm to find the maximum agreement. In particular, we assume the marginal distributions as fixed and we consider all multivariate tables with fixed one-way margins. The proposed algorithm can be applied with a general weighting scheme, not limited to linear or quadratic. Notice that the problem can be tackled also within the theory of integer linear programming (maximize the kappa statistics taking fixed the margins), but Markov moves provide a flexible tool and a solution easy to explain.

The paper is organized as follows. In Sect. 1 we recall the notation and the basic definitions about the Cohen’s $\kappa$, the weighted Cohen’s $\kappa _w$, and the Conger’s $\kappa _C$ and $\kappa _{C,w}$ for the multi-rater case. In Sect. 2, we compute the Markov bases for the rater agreement problem in the two-rater and in the multi-rater cases. Such Markov bases are used in Sect. 3 to state some results on the structure of the configuration of maximum agreement in connection with the (metric) properties of the weighting schemes. Section 4 is devoted to the illustration of a simulated annealing algorithm to actually find the configuration of maximum agreement, while in Sect. 5 the results of a simulation study are presented and discussed. Finally, Sect. 6 contains some concluding remarks and pointers to future directions.

## Notation and Basic Recalls

In this section, we briefly review the basic definitions about the kappa-type indices of agreement which will be used in the paper. We first focus on the two-rater setting.

Let us consider the ratings of the two raters as a pair of random variables *X* and *Y* on the set $\{1, \ldots , k\}$, or more generally on a finite ground set $\{x_1, \ldots , x_k\}$. Let us denote with $p_{ij}$ the probability of the cell (*i*, *j*), and with $p_{i+}$ ($i=1, \ldots , k$) and $p_{+j}$ ($j=1, \ldots , k$) the marginal distributions of *X* and *Y*, respectively. The Cohen’s $\kappa$ is defined as:

1 $$\begin{aligned} \kappa = \frac{\sum _{i=1}^k p_{ii} - \sum _{i=1}^k p_{i+}p_{+i}}{1 - \sum _{i=1}^k p_{i+}p_{+i}} = 1 - \frac{\sum _{(i,j) \in D} p_{ij}}{\sum _{(i,j) \in D} p_{i+}p_{+j}} \, , \end{aligned}$$

where $D=\{(i,j) \ : \ i \ne j\}$ is the set of the disagreement cells.

Given a matrix of weights of agreement $W=(w_{ij})$ with $0 \le w_{ij} < 1$ for all *i*, *j* with $i \ne j$, and $w_{ii}=1$ for all *i*, the weighted kappa is:

2 $$\begin{aligned} \kappa _w = \frac{\sum _{i,j=1}^k w_{ij}p_{ij} - \sum _{i,j=1}^k w_{ij}p_{i+}p_{+j}}{1 - \sum _{i,j=1}^k w_{ij}p_{i+}p_{+j}} = 1 - \frac{\sum _{(i,j) \in D} u_{ij} p_{ij}}{\sum _{(i,j) \in D} u_{i,j}p_{i+}p_{+j}} \, , \end{aligned}$$

where in the second expression $u_{ij}=1-w_{ij}$. Although not strictly necessary for the theory of rater agreement, we suppose that the matrices *W* and $U=(u_{ij})$ are symmetric, because some of our results are based on the properties of the metric functions, where symmetry is one the axioms. In the previous formulas, the $u_{ij}$ are weights of disagreement, and it is easily seen that $u_{ij}=0$ on the main diagonal and $0<u_{ij}\le 1$ for $i \ne j$. When a sample is available, the indices $\kappa$ and $\kappa _w$ are estimated by replacing in Eqs. (1) and (2) the theoretical probabilities with the corresponding sample proportions. On a sample of size *N*, we denote with $n_{ij}$ the count of the cell (*i*, *j*) and therefore sample proportion is ${\hat{p}}_{ij}=n_{ij}/N$.

Among the most commonly used weighting schemes there are:

- the quadratic weights (see Fleiss and Cohen 1973):
  3 $$\begin{aligned} u_{ij} = \frac{(i-j)^2}{(k-1)^2} \end{aligned}$$
- the linear weights (see Cicchetti and Allison 1971):
  4 $$\begin{aligned} u_{ij} = \frac{|i-j|}{k-1} \end{aligned}$$

Moreover, the unweighted $\kappa$ in Eq. (1) can be considered as a special case of the weighted $\kappa _w$ by setting

5 $$\begin{aligned} u_{ij} = \left\{ \begin{array}{ll}0 \ &{} \ \text{ for } i=j \\ 1 \ &{} \ \text{ otherwise } \end{array}\right. \end{aligned}$$

Recent discussions on the choice, use, and interpretation of the different weighting schemes can be found in Warrens (2013) and Kvålseth (2018). On one side, the main reasons in favor of the quadratic and linear weights are essentially of theoretical nature. In fact, the quadratic weights lead to the interpretation of the weighted kappa as the intraclass correlation coefficient, see Schuster (2004). On the other side, the linear weights allow us to define the weighted kappa as a weighted average of kappas for the $2 \times 2$ tables obtained by collapsing adjacent categories, see Vanbelle and Albert (2009). However, undesirable behaviors of the weighted kappa for some data set can be observed under both choices of the weights, and thus the interpretation of the value of kappa is not easy in general. We will come back to this issue later in the paper, when we will use Markov moves to find the maximum agreement. Another interesting interpretation of the linear and quadratic weights is discussed in Li (2016), where matrix *W* is decomposed into a sum of suitable rank one matrices.

In order to illustrate our theory, we also consider a square-root version of the weights, namely:

6 $$\begin{aligned} u_{ij} = \frac{\sqrt{|i-j|}}{\sqrt{k-1}} \end{aligned}$$

As a preliminary remark, notice that the linear weights in Eq. (4) and the square-root weights in Eq. (6) define a distance in ${{\mathbb {R}}}$, while the quadratic weights in Eq. (3) do not, because the triangular inequality is not satisfied. Usually, functions like the quadratic weights are called dissimilarities. In this paper, when the matrix *U* is a distance matrix, we name the weights as “distance weights,” and in particular, we refer to the weights in Eq. (6) as to the sqrt weights. Moreover, we use the notation $\kappa _q$, $\kappa _l$, $\kappa _s$ when quadratic, linear, or sqrt weights are used, while we denote with $\kappa _w$ the kappa with a general weight.

Observe that the distance defined by the linear weights is the usual Euclidean distance in ${{\mathbb {R}}}$, and it has a special behavior in terms of the triangular inequality. In fact, for $i<j<h$ the triangular inequality becomes an equality: $u_{ih}=u_{ij}+u_{jh}$. We will exploit this property later in the paper.

In the multi-rater setting, we consider the ratings of *r* raters as *r* random variables $X_1, \ldots , X_r$ on the same set $\{1, \ldots , k\}$, or more generally on $\{x_1, \ldots , x_k\}$. The observed data form a $k^r$ table. We denote with $p_{i_1 \ldots i_r}$ the probability of the cell $(i_1, \ldots , i_r)$, and with $n_{i_1 \ldots i_r}$ the corresponding observed count on a sample of size *N*. Moreover, we denote with $p^{(u)}$ the one-dimensional marginal distribution of $X_u$, and with $p^{(uv)}$ the two-dimensional marginal distribution of the pair $(X_u,X_v)$.

To measure the agreement in the multi-rater setting, it is customary to use the Conger’s $\kappa _C$, originally introduced in Conger (1980) and the re-analyzed in several papers, see, e.g., Vanbelle (2019). The Conger’s $\kappa _c$ is based on a pairwise rater agreement analysis. It is defined as:

7 $$\begin{aligned} \kappa _C = \frac{p_o - p_e}{1 - p_e} \end{aligned}$$

where $p_o$ is the mean proportion of agreement between all $r(r-1)/2$ pairs of raters, and similarly $p_e$ is the mean proportion of expected agreement between all $r(r-1)/2$ pairs of raters under independence. In formulas,

8 $$\begin{aligned} p_o = \frac{2}{r(r-1)} \sum _{u,v \in \{1, \ldots , r\},u<v} \sum _{i=1}^k p^{(uv)}_{ii} \end{aligned}$$

and

9 $$\begin{aligned} p_e = \frac{2}{r(r-1)} \sum _{u,v \in \{1, \ldots , r\},u<v} \sum _{i=1}^k p^{(u)}_{i}p^{(v)}_{i} \, . \end{aligned}$$

Since the Conger’s $\kappa _C$ is based on the two-way margins of the $k^r$ table, it is easy to define a weighted version of the Conger’s kappa as follows:

10 $$\begin{aligned} \kappa _{C,w} = \frac{p_{o,w} - p_{e,w}}{1 - p_{e,w}} \end{aligned}$$

with

11 $$\begin{aligned} p_{o,w} = \frac{2}{r(r-1)} \sum _{u,v \in \{1, \ldots , r\},u<v} \sum _{i,j=1}^k w_{ij}p^{(uv)}_{ij} \end{aligned}$$

and

12 $$\begin{aligned} p_{e,w} = \frac{2}{r(r-1)} \sum _{u,v \in \{1, \ldots , r\},u<v} \sum _{i,j=1}^k w_{ij}p^{(u)}_{i}p^{(v)}_{j} \end{aligned}$$

In the above definition, the weights are the same for all pairs *u*, *v* of raters, but the definition can be easily extended to the case of different weights on different two-way margins. Also notice that the Conger’s $\kappa _C$ can be defined in the general case of *g*-wise agreement, as in the original paper Conger (1980). This is done by taking the $\kappa _{C}$ unchanged in Eq. (7), and computing the observed agreement and the expected agreement in Eqs. (8) and (9) on the *g*-way marginal tables instead of the two-way tables. However, when the weighted version $\kappa _{C,w}$ in Eqs. (10)–(12) is considered, the pairwise agreement is the most reasonable choice, and the extension to the *g*-wise agreement would require new definitions of the weighting schemes.

## Markov Bases

In this section, we introduce the main tools from algebraic statistics needed in our framework. In particular, we define the notion of Markov basis and we compute the relevant Markov bases for the rater agreement problems.

Let *n* be an observed contingency table, possibly multi-way. An integer-valued statistic is a function $T:{{\mathbb {N}}}^{k^r} \longrightarrow {{\mathbb {N}}}^s$. Since we need to compute the maximum agreement with fixed marginal distributions we are particularly interested in the function

13 $$\begin{aligned} T : n \longmapsto ((n_{i+})_{i=1,\ldots ,k},(n_{+j})_{j=1,\ldots ,k}) \end{aligned}$$

in the two-way case and

14 $$\begin{aligned} T : n \longmapsto ((n^{(1)}_i)_{i=1,\ldots ,k}, \ldots , (n^{(r)}_i)_{i=1,\ldots ,k}) \end{aligned}$$

in the general multi-rater case, where $n^{(s)}_i$ is the *i*-th entry of the marginal distribution of the *s*-th rater.

### Definition 1

Given a statistic *T*, the fiber (or reference set) of a contingency table *n* is the set

15 $$\begin{aligned} {\mathcal {F}}_T(n) = \{ n' \in {{\mathbb {N}}}^{k^r} \ | \ T(n') = T(n) \} \, . \end{aligned}$$

### Definition 2

A Markov move for the statistic *T* is an integer-valued table *m* such that $T(m)=0$.

### Definition 3

A Markov basis for the fiber ${{\mathcal {F}}}_T(n)$ of a table *n* is a set of Markov moves

$$\begin{aligned} {{\mathcal {M}}}_{n,T} = \{m^{(1)}, \ldots , m^{(\ell )} \} \end{aligned}$$

such that for each pair of tables $n',n'' \in {{\mathcal {F}}}_T(n)$ there exists a sequence of moves $(m^{(i_1)}, \ldots , m^{(i_Q)})$ such that

1. $$n'' = n' + \sum _{j=1}^Q m^{(i_j)}$$
2. $$n' + \sum _{j=1}^q m^{(i_j)} \ge 0$$ for all $$q=1, \ldots ,Q$$.

In words, a Markov basis is a set of moves which makes the fiber ${{\mathcal {F}}}_T(n)$ connected and all intermediate steps are nonnegative. In algebraic statistics, this is the main tool to define a Metropolis-like Markov chain Monte Carlo algorithm for doing exact inference for contingency tables. For a comprehensive introduction to Markov bases and their use in statistics, the reader can refer to the books Sullivant (2018) and Aoki et al. (2012).

Since we are particularly interested in the computation of the maximum agreement given the marginal distributions, we need the Markov bases for the statistic *T* in Eqs. (13) and (14).

Following Diaconis and Sturmfels (1998), in the general case the computation of a Markov basis needs symbolic computation and is actually not feasible for large-sized tables. However, the Markov bases for the fibers considered in this paper can be theoretically characterized and therefore no symbolic computation is involved. For an overview on the computation of Markov bases through symbolic software, the underlying computational problems, and the actual limitations for large tables, the reader can refer to Aoki et al. (2012).

As a first step, we recall a result from Diaconis and Sturmfels (1998) about the Markov basis for two-way tables with fixed margins.

### Definition 4

Let $i,i'$ be two distinct row indices and $j,j'$ be two distinct column indices. A basic move is a move *m* such that

$$\begin{aligned} m_{ij}=m_{i'j'}=+1, \qquad m_{ij'}=m_{i'j}=-1 \end{aligned}$$

and is 0 otherwise.

**Fig. 2:**
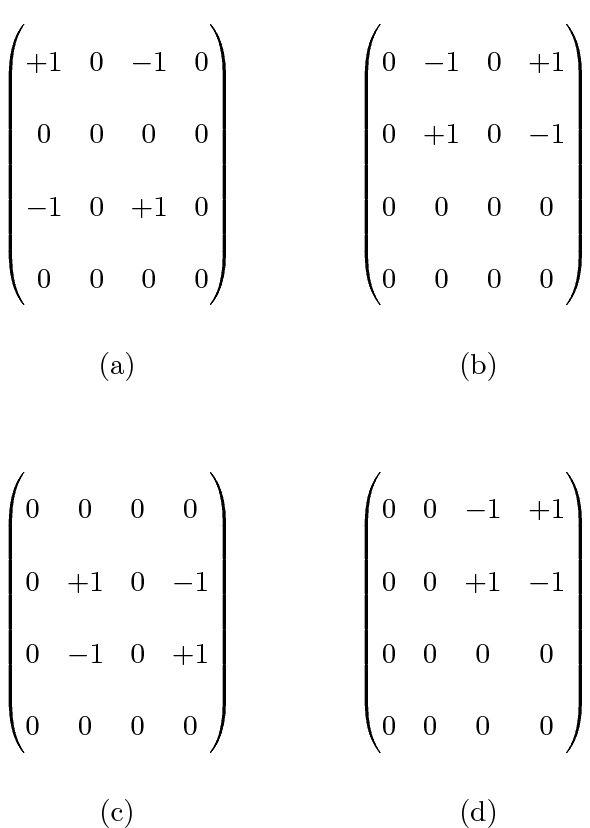
Four basic moves for the two-rater problem. **a** Two nonzero elements on the diagonal; **b** one nonzero element on the diagonal, the move lies on the upper triangle; **c** one nonzero element on the diagonal, the move lies on both the upper and the lower triangle; **d** no nonzero elements on the diagonal.

Some examples of basic moves in the case of 4 categories are given in Fig. 2. Such moves have different behavior in terms of agreement. We will discuss all these types of moves in the next section.

### Proposition 1

The set of basic moves in Definition 4 is a Markov basis for the fiber in Eq. (15) for the two-rater problem.

The basic moves in the multi-rater setting are defined by extending the previous definition to more than two dimensions. Informally, one takes two $+1$’s in two cells with at least two distinct coordinates and then arranges the $-1$’s in order to have the correct projections in all two-way margins. More formally, we can state the following definition.

### Definition 5

A basic move *m* for the multi-rater problem is a table with 4 nonzero entries:

- *m* is equal to $+1$ in $(i_1, \ldots i_r)$ and in $(i'_1,\ldots , i'_r)$ with at least two different indices. Without loss of generality, suppose that the distinct indices are $i_1, \ldots , i_q$, $q \ge 2$;
- *m* is equal to $-1$ in $(j_1,\ldots , j_q,i_{q+1},\ldots , i_r)$ and in $(j'_1,\ldots , j'_q,i_{q+1},\ldots , i_r)$ with
  (i) $$j_s=i_s$$, $$j'_s=i'_s$$ for $$s \in {{\mathcal {S}}}$$
  (ii) $$j_s=i'_s$$, $$j'_s=i_s$$ for $$s \notin {{\mathcal {S}}}$$
  where ${{\mathcal {S}}}$ is a non-empty subset of $\{1, \ldots , q\}$.

**Fig. 3:**
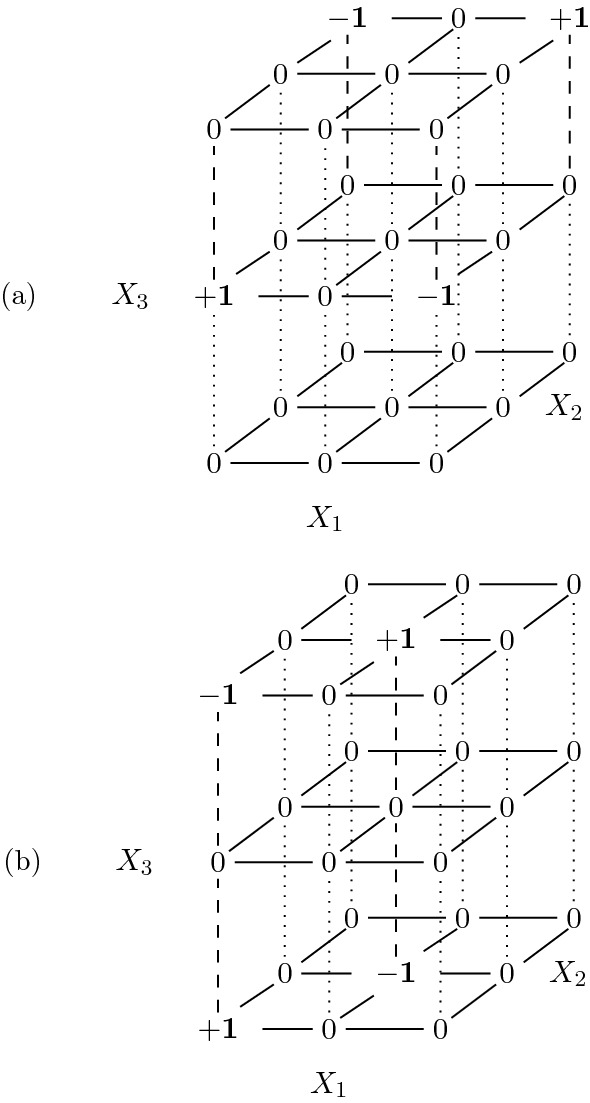
Two basic moves for the three-rater problem. A move of type (**a**) and a move of type (**b**) from Proposition 2.

It is easy to see that this definition reduces to Definition 4 when $r=2$. Two examples of basic moves in the $3^3$ case are illustrated in Fig. 3.

The fact that basic moves are enough to connect the fiber in Eq. (15) can be derived from the theory of toric fiber products to be found in Sullivant (2007). This allows us to avoid symbolic computations and to make available the relevant Markov bases also for large-sized tables.

### Proposition 2

The set of basic moves in Definition 5 is a Markov basis for the fiber in Eq. (15) when $r>2$.

### Proof

First, note that all basic moves in Definition 5 are in the kernel of the marginalization map *T*.

Since for $r=2$ the basic moves in Definition 5 coincide with the basic moves in 4, the result is true when $r=2$. We proceed by induction on *r*. Let us suppose that the result holds for $r-1$ raters, and we prove it for *r* raters. We apply Theorem 13 in Sullivant (2007). Given a Markov basis ${{\mathcal {M}}}_{r-1}$ for the problem with $(r-1)$ raters, a Markov basis ${{\mathcal {M}}}_{r}$ for the problem with *r* raters is the union of the following sets of moves

- split each move of ${{\mathcal {M}}}_{r-1}$ by putting one $+1$ and one $-1$ at a given level *h* of $X_r$ ($h=1, \ldots , k$) and the other $+1$ and $-1$ at a level $h'$ of $X_r$ ($h'=h, \ldots , k$);
- for any two distinct cells $(i_1, \ldots , i_{r-1})$ and $(i'_1, \ldots , i'_{r-1})$ on the $(r-1)$-dimensional table, and for any two distinct levels $h,h'$ of $X_r$, take the move with $+1$ in $(i_1, \ldots , i_{r-1},h)$ and in $(i'_1, \ldots , i'_{r-1},h')$ and with $-1$ in $(i_1, \ldots , i_{r-1},h')$ and in $(i'_1, \ldots , i'_{r-1},h)$.

Since all the moves defined in items (*a*) and (*b*) above are basic moves, the result is proved. $\square$

As noticed in Introduction, Markov bases in algebraic statistics are usually defined in algebraic statistics in order to perform exact tests with a Metropolis–Hastings algorithm, and therefore to generate all contingency tables with the same value of the sufficient statistics as the observed table. Here, we simply use Markov bases to compute all the tables with fixed margins.

## The Effect of the Markov Moves on the Kappa Indices

Now, we use the basic moves of the Markov bases in order to better understand the meaning of the weighted kappa. The basic idea is to apply the definition of Markov basis to analyze the rater agreement in connection with the weighting schemes. We show here that most of the basic moves have a precise behavior in terms of their effect on the kappa indices and therefore we analyze how the rater agreement changes when a Markov move is applied. Moreover, the configuration of maximum agreement can be reached with a finite number of Markov moves, starting from the observed table, and the analysis with basic moves helps us in understanding the structure of the configurations with maximum agreement.

Since our analysis is performed with fixed marginal distributions, the kappa indices are monotonic with the observed agreement. So, to ease the formulas, we consider the quantities

16 $$\begin{aligned} A_{o,w}(n) = \frac{1}{N} \sum _{i,j=1}^k w_{ij}n_{ij} \end{aligned}$$

in the two-rater setting, and

17 $$\begin{aligned} A_{o,w}(n) = \frac{2}{r(r-1)} \sum _{u,v \in \{1, \ldots , r\},u<v} \frac{1}{N} \sum _{i,j=1}^k w_{ij}n^{(uv)}_{ij} \end{aligned}$$

in the multi-rater setting.

Let us start with some results in the two-rater setting.

### Lemma 1

Let *n* be an observed agreement table, let $i \ne j$ be two indices, and let *m* be the basic move with

$$\begin{aligned} m_{ii}=m_{jj}=+1, \qquad m_{ij}=m_{ji}=-1 \, . \end{aligned}$$

If $n_{ij}>0$ and $n_{ji}>0$, then

18 $$\begin{aligned} A_{o,w}(n+m) \ge A_{o,w}(n) \, . \end{aligned}$$

### Proof

From Eq. (16) and using the disagreement weights, we get

$$\begin{aligned} A_{o,w}(n+m) - A_{o,w}(n) = A_{o,w}(m) = - \frac{1}{N} \sum _{i,j=1}^k u_{ij}m_{ij} = \frac{2}{N} u_{ij} \ge 0 \, . \end{aligned}$$

$$\square $$

Lemma 1 is valid for all weighting schemes and tells us that if there are positive counts in symmetric cells, then it is always possible to construct an observed table with higher observed agreement by applying a simple move. This is quite intuitive, since Eq. (18) roughly says that moving counts on the diagonal increases the observed agreement.

Nonetheless, apart from the symmetric basic moves as displayed in Fig. 2a, for the other types of basic moves there is not a common behavior in terms of observed agreement. Remember that, earlier in the paper, we have noticed that some weighting schemes defines a distance on the ground set $\{x_1, \ldots , x_k\}$ while other schemes do not. The following proposition states a partial result when only one cell of the diagonal is involved in the basic move.

### Proposition 3

Let *n* be an observed agreement table, let $i< j < h$ be three indices, and let *m* be the basic move with

$$\begin{aligned} m_{ih}=m_{jj}=+1, \qquad m_{ij}=m_{jh}=-1 \, . \end{aligned}$$.

If $n_{ij}>0$ and $n_{jh}>0$ and a distance weighting scheme is used, then

$$\begin{aligned} A_{o,w}(n+m) \ge A_{o,w}(n) \, . \end{aligned}$$

The same holds if $i>j>h$.

### Proof

Let us consider the case $i< j < h$. (The other case has a similar proof.)

From Eq. (16) and using the disagreement weights, we get

$$\begin{aligned} A_{o,w}(n+m) - A_{o,w}(n)= & {} A_{o,w}(m) = - \frac{1}{N} \sum _{i,j=1}^k u_{ij}m_{ij} = \\= & {} \frac{1}{N} \left( u_{ij} + u_{jh}- u_{ih} \right) \ge 0 \end{aligned}$$

by virtue of the triangular inequality. $\square$

In Proposition 3, the move *m* has one nonzero element on the diagonal. In the case $i< j < h$ the move lies in the upper triangle of the table, while in the case $i>j>h$ lies in the lower one.

Some remarks are now in order. First, note that in Proposition 3 the assumption of distance weights is essential. For weighting schemes derived or not derived from a distance we observe opposite behaviors of the weighted kappa. For instance, let us consider the observed table below:

$$\begin{aligned} n=\begin{pmatrix} 4 &{}\quad 0 &{}\quad 0 &{}\quad 0 \\ 0 &{}\quad 4 &{}\quad 1 &{}\quad 0 \\ 0 &{}\quad 0 &{}\quad 4 &{}\quad 1 \\ 0 &{}\quad 0 &{}\quad 0 &{}\quad 4 \end{pmatrix} \, . \end{aligned}$$

We can apply the move

$$\begin{aligned} m=\begin{pmatrix} 0 &{}\quad 0 &{}\quad 0 &{}\quad 0 \\ 0 &{}\quad 0 &{}\quad -1 &{}\quad +1 \\ 0 &{}\quad 0 &{}\quad +1 &{}\quad -1 \\ 0 &{}\quad 0 &{}\quad 0 &{}\quad 0 \end{pmatrix} \end{aligned}$$

and we obtain

$$\begin{aligned} n'=n+m=\begin{pmatrix} 4 &{}\quad 0 &{}\quad 0 &{}\quad 0 \\ 0 &{}\quad 4 &{}\quad 0 &{}\quad 1 \\ 0 &{}\quad 0 &{}\quad 5 &{}\quad 0 \\ 0 &{}\quad 0 &{}\quad 0 &{}\quad 4 \end{pmatrix} \, . \end{aligned}$$

Comparing the value of $\kappa _w$ of *n* and $n'$ we note that:

- With a distance weight we have $\kappa _w(n')>\kappa _w(n)$ by virtue of Proposition 3;
- With quadratic weights we have $\kappa _q(n')<\kappa _q(n)$;
- With linear weights we get $\kappa _l(n')=\kappa _l(n)$.

While with distance weights the maximum agreement is achieved by maximizing the counts in the diagonal cells, with quadratic weights a certain amount of moderate disagreement is preferred to a small amount of strong disagreement.

Moreover, from the above example, we observe there is a special behavior of the linear weights, because some Markov moves do not change the value of the weighted kappa. This affects also the problem of finding the configuration with maximum agreement, since in general such a configuration is not unique. We state below a result for linear weights, and we will discuss this issue in the next section from the point of view of computations.

### Proposition 4

Let us consider four indices $i_1<i_2\le j_1 <j_2$ or $j_1< j_2 \le i_1 < i_2$, and take the basic move *m* with $m_{i_1j_1}=m_{i_2j_2}=+1$ and $m_{i_1j_2}=m_{i_2j_1}=-1$. If *n* is a table with $n_{i_1j_2}>0$ and $n_{i_2j_1}>0$. Using the linear weights we get

$$\begin{aligned} \kappa _l(n) = \kappa _l(n+m) \, . \end{aligned}$$

### Proof

As in the previous proposition, let us consider only the case $i_1<i_2\le j_1 <j_2$.

Notice that the conditions $n_{i_1j_2}>0$ and $n_{i_2j_1}>0$ are needed in order to have a nonnegative table $n'=n+m$. Since *n* and $n'$ have the same margins, it is enough to compare the observed agreement. From Eq. (17), we get:

$$\begin{aligned} A_{o,w}(n')-A_{o,w}(n)= & {} A_{o,w}(m) = - \frac{1}{N} \sum _{i,j=1}^k, u_{ij}m_{ij} \\= & {} -\frac{1}{N} \cdot \frac{(j_1-i_i)+(j_2-i_2)-(j_2-i_1)-(j_1-i_2)}{k-1} = 0 \, . \end{aligned}$$

$$\square $$

The condition $i_1<i_2\le j_1 <j_2$ means that we apply a move on one side of the table w.r.t. the diagonal, and one nonzero element of the move is on the diagonal when $i_2=j_1$. Under such a condition, the move does not affect the value of the weighted kappa.

**Fig. 4:**
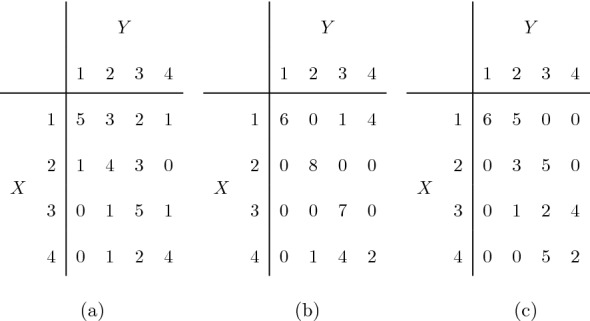
A synthetic observed table (**a**) and two tables with the same margins and with the same weighted kappa under linear weights (**b**, **c**).

In view of Proposition 4, the uniqueness of the table with a given value of $\kappa _w$ is not guaranteed under any of weighting schemes, but this issue is especially relevant for the linear weights. To illustrate this, let us consider the table (with synthetic data) in Fig. 4a. By direct enumeration of the 644, 850 tables of the fiber, one finds 1527 tables with the same margins and with the same value of the weighted kappa with linear weights as the observed table, i.e., $\kappa _l=0.5023$. Among those tables, the weighted kappa with quadratic weights ranges from $\kappa _q=0.3774$ to $\kappa _q=0.7406$. The minimum is achieved in 3 tables, one of which is in Fig. 4b, while the maximum is achieved in 3 tables, one of which is in Fig. 4c.

Let us now turn to the multi-rater setting. From Eqs. (10), (8), (9) it is easy to argue that the effect of a basic move on the value of the Conger’s $\kappa _C$ is yielded by the two-way margins of the move. Each two-way projection applies to a two-way margin and gives its own contribution in the sum in Eq. (8).

The following proposition collects the properties of the two-way margins of a basic move, and its proof is immediate.

### Proposition 5

Let *m* be a basic move in the multi-rater case with *r* raters. Suppose that *m* is equal to $+1$ in $(i_1, \ldots ,i_r)$ and in $(i'_1,\ldots , i'_r)$ and is equal to $-1$ in $(j_1, \ldots ,j_r)$ and in $(j'_1, \ldots , j'_r)$. The projection of *m* on the pair (*U*, *V*) is:

- a basic move for the two-way problem, if the four pairs $(i_u, i_v)$, $(i'_u,i'_v)$, $(j_u,j_v)$, $(j'_u,j'_v)$ are all distinct;
- a null move, otherwise.

Note that, following the definition of basic move in Definition 5, it is easy to see that a multi-rater basic move *m* always yields at least one basic move on some two-dimensional margin.

For example, both the moves for the three-rater problem displayed in Fig. 3 produce a basic move on two two-way margins and a null move on one margin.

In general, the analysis of the effect of the basic moves on the Conger’s $\kappa _C$ is more difficult than in the two-rater case. Nevertheless, we can state the following lemma, which generalizes Lemma 1.

### Lemma 2

Let $i \ne j$ be two indices, and let *m* be a basic move with

$$\begin{aligned} m_{i\ldots i}=m_{j\ldots j}=+1 \, . \end{aligned}$$

If $n+m$ is nonnegative, then

19 $$\begin{aligned} A_{o,w}(n+m) \ge A_{o,w}(n) \, . \end{aligned}$$

### Proof

It is enough to observe that all two-way margins of *m* are either a null move or a basic move satisfying the hypothesis of Lemma 1. $\square$

**Fig. 5:**
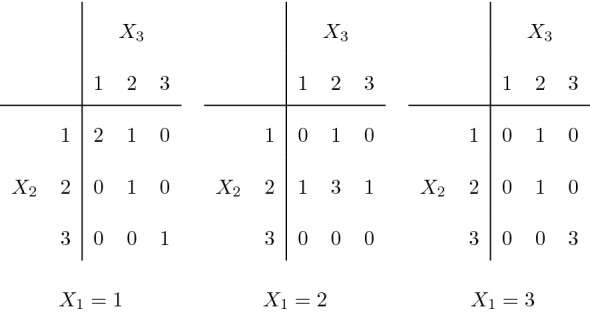
An observed table with 3 raters and 3 levels.

Also the multi-rater case the issue of non-uniqueness is especially relevant under linear weights. For instance, let us consider the three way table in Fig. 5. Although the table is rather sparse (the sample size is 16 in a contingency table with 27 cells), there are 2, 324 tables with the same value of $\kappa _{C,l}=0.4872$. Under quadratic weights, such tables yield values of $\kappa _{C,q}$ ranging from 0.3364 to 0.6313.

## Simulated Annealing for Maximum Agreement

In this section, we show how to use a simulated annealing algorithm to determine the maximum value of the weighted kappa with fixed marginal distributions and to find a table where the maximum is actually reached. The Markov bases introduced in Sect. 2 are used in the algorithm to define the neighbors of the contingency tables and to navigate the fiber of an observed table.

While the computation of the maximum agreement is simple for the unweighted $\kappa$ in the two-rater setting, the problem is not trivial when the weighted $\kappa _w$ is considered, or we use the Conger’s $\kappa _C$ or $\kappa _{C_w}$ for the multi-rater problem.

The Markov chain simulated annealing algorithm starts from the observed table and runs at each step *b* ($b=1, \ldots , B$) as follows. First, we choose a move *m* in the relevant Markov basis ${{\mathcal {M}}}$ and we define $n'=n+m$; if $n'$ is a nonnegative table, then we move the chain from *n* to $n'$ with a transition probability depending on two factors. On the one hand, the transition probability is equal to 1 if the move causes an increase in the observed agreement, while it is less than one if the move causes a decrease in the observed agreement, and this probability is lower the more the decrease is high. On the other hand, the transition probability decreases with the time.

In practice, in the first part of the walk the Markov chain performs exploration, while in the second part it performs exploitation, because the probability of an actual move toward a table with smaller observer agreement decreases with the time. With our notation, the formula for the transition probability is:

$$\begin{aligned} \min \left\{ \exp ((A_{o,w}(n')-A_{o,w}(n'))/\tau _b), 1 \right\} , \end{aligned}$$

where $A_{o,w}$ is the observed agreement in the table *n*, and $\tau _b$ is the temperature at time *b*.

**Fig. 6:**
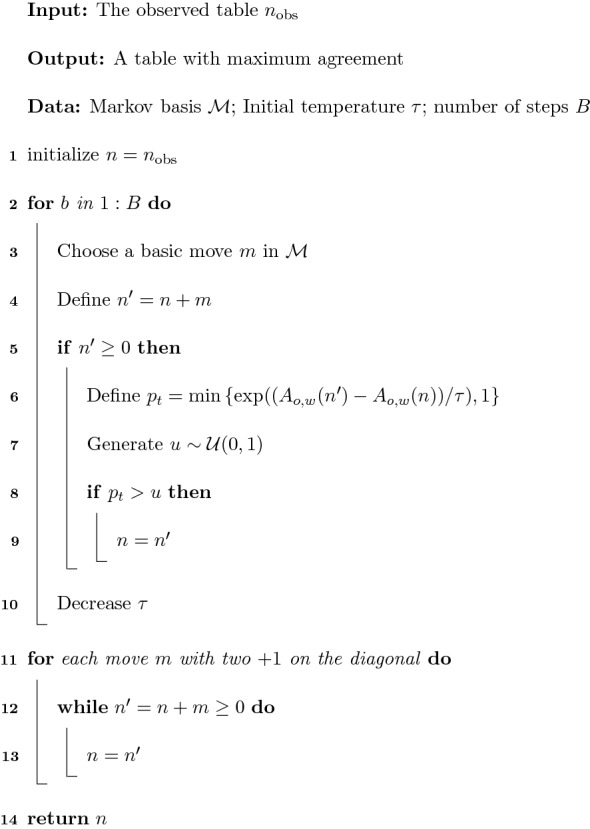
Simulated annealing for maximum agreement.

As a special feature of this algorithm, we have added a final step to apply all possible moves with two $+1$ on the main diagonal, thus exploiting the results in Lemmas 1 and 2. The pseudo-code of the algorithm is in Fig. 6.

The reader can refer to Suman and Kumar (2006) for a general introduction to simulated annealing in the discrete case and for a discussion on the computational details of the algorithm, as, for instance, the choice of the temperature function $\tau _b$. In particular, from our experiments, the choice of the function for the temperature decrease does not affect the performance of the algorithm, and thus, we have used a temperature of the form $\tau =\tau _0 \cdot d^b$.

**Fig. 7:**
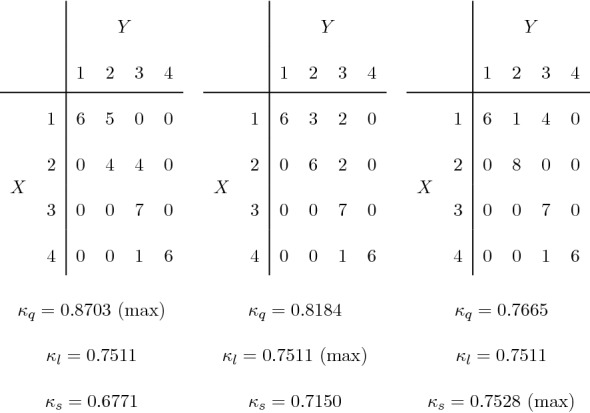
Configurations with maximum weighted kappa for the observed table in Fig. 4 with quadratic weights (left), linear weights (center), sqrt weights (right).

Notice that the non-uniqueness of the configuration is still an issue also when finding the maximum, especially using the linear weights. As an example in the two-rater framework, consider again the observed table in Fig. 4a. With linear weights, there are 5 tables which reach the maximum value of $\kappa _l=0.7511$, and among these tables the $\kappa _q$ ranges from 0.7665 to 0.8703, the latter being also the maximum with quadratic weights. The maximum with the sqrt weights is $\kappa _s=0.7528$. The three configurations obtained with our algorithm are displayed in Fig. 7. In accordance with the findings in the previous sections, we note that quadratic weights avoid strong disagreement cells, while sqrt weights fill the diagonal as much as possible. Again, the table with maximum linear weight is not unique, and in fact, the three tables in Fig. 7 share the same of $\kappa _l$.

The algorithm converges very fast, at least for small- and medium-sized tables, yielding the maximum value of the weighted kappa and a table where such a maximum is reached in less than 1 second on a standard PC. For large tables, the convergence takes long times, and the problem becomes fast unfeasible when the number of cells is large. In fact, on the one side large tables are usually sparse, on the other side, the relevant Markov basis is large, and at each step, the probability of an applicable move is very low. As a consequence, for large tables the number of Markov chain steps *B* must be quite large to ensure convergence. Some experiments are shown through a simulation study in the next section. In our experiments, we have found a fast convergence: for instance, on a standard PC the algorithm for the two-rater problem runs in less than 1 s for tables up to $k=10$ rating categories (100 cells), and in less than 10 s for tables up to $k=18$ rating categories (324 cells).

Note that one can replace the fixed run length *B* with a stopping rule, and this is the strategy implemented in our simulation study. For instance, in small problems one can stop the algorithm when the algorithm does produce actual moves for 1000 consecutive steps. For large tables, the stopping rule must take into account also the cardinality of the Markov basis. More details on this point are discussed in the next section.

In general, the use of algebraic statistics in the case of large tables is problematic, and the curse of dimensionality is a known issue of this discipline. The definition of new techniques to speed up the convergence of Markov chain-based algorithms within algebraic statistics is still a current research topic, see, for instance, Windisch (2016), and only ad hoc solutions for special problems are currently available.

## Simulation Study

In order to show the practical applicability of the algorithm introduced in the previous section, and to study its convergence properties, we have designed and performed a simulation study with several scenarios. For the two-rater case, we have considered three values of the number of levels *k* ($k=3,5,7$) and two sample sizes ($N=20,100$). Moreover, two types of marginal distributions are considered: a first case with homogeneous uniform margins and a second case with non-homogeneous margins. In the first case, the tables are generated from a multinomial distribution with probabilities given by $\mu \otimes \mu$, with $\mu =(1/k, \ldots , 1/k)$, while in the second case the probability parameter of the multinomial distribution is $\mu \otimes \nu$ with $\mu \ne \nu$. In the non-homogeneous case, the parameters $\mu$ and $\nu$ are chosen to account for the tendency of a rater to choose rating levels higher or lower than those of the other rater. For instance, in the $3 \times 3$ case, we have used $\mu =(2/5,2/5,1/5)$ and $\nu =(1/5,2/5,2/5)$.

Notice that, with this procedure, we obtain different observed marginal distributions also when the parameter of the multinomial distribution is of the form $\mu \otimes \mu$, and thus, the problem of finding the maximum weighted kappa is not trivial even in these scenarios. Also a simulation study for the three-rater case is presented, but limited to two numbers of categories $k=3,5$.

The convergence of the algorithm is measured as follows. The algorithm stops when there is a sufficiently large number *c* of consecutive steps with no change in the observed agreement (and therefore without changes in the weighted kappa). The number *c* must take into account the number of moves in the Markov basis. We have defined here $c=\max \{10\cdot \#{{\mathcal {M}}}; 1,000\}$. This choice of *c* is a reasonable trade-off between accuracy and speed. For each scenario, a sample of 1, 000 tables is generated and the distribution of the stopping time *ST* is approximated through the 1, 000 observed values. The simulation study has been performed using three weighting schemes: quadratic, linear, and sqrt.

The results are displayed in Table 1 for the two-rater scenarios and in Table 2 for the there-rater scenarios. The mean, the standard deviation, and the 99th percentile of the convergence time *ST* are reported. In such tables, only the results for tables with homogeneous margins are considered. Since the results for tables with non-homogeneous margins are very similar, they are reported as Tables 3 and 4 in “Appendix.”

**Table 1:** Two-rater case with homogeneous marginal distributions.

Weight	*k*	*N*	Mean	sd	$q_{0.99}$
Quadratic	3	20	1064.8	32.0	1164
		100	1264.5	75.2	1481
	5	20	2568.5	277.7	3418
		100	3943.6	637.9	5922
	7	20	11,300.4	1157.9	14,914
		100	15,497.8	2211.2	21,997
Linear	3	20	1049.4	25.3	1126
		100	1170.8	47.6	1307
	5	20	2407.3	249.1	3233
		100	3064.6	428.6	4420
	7	20	10,532.5	1042.5	13,784
		100	12,358.5	1516.0	16,941
Sqrt	3	20	1055.6	27.3	1139
		100	1165.5	44.6	1290
	5	20	2499.6	257.1	3298
		100	3088.3	401.9	4318
	7	20	10,955.3	1018.3	14,054
		100	12,872.3	1539.2	18,235

Time to convergence (mean, standard deviation and 99th percentile) of the simulated annealing algorithm for different numbers of levels *k* and sample sizes *N*.

**Table 2:** Three-rater case with homogeneous marginal distributions.

Weight	*k*	*N*	Mean	sd	$q_{0.99}$
Quadratic	3	20	4227.8	491.8	5778
		100	5753.2	830.9	8237
	5	20	115,590.4	11,611.1	153,122
		100	142,858.7	17,776.5	190,784
Linear	3	20	4057.9	402.7	5619
		100	5075.6	629.0	6987
	5	20	110,198.5	10,633.9	146,114
		100	124,430.1	13,703.3	167,849
Sqrt	3	20	4213.5	508.0	5842
		100	5248.2	754.4	7760
	5	20	117,436.7	12,774.8	159,911
		100	131,752.7	15,281.4	178,512

Time to convergence (mean, standard deviation and 99th percentile) of the simulated annealing algorithm for different numbers of levels *k* and sample sizes *N*.

From the results, we see that the time to convergence increases with the sample size and with the dimension of the table, and this is particularly relevant in the three-rater case. As discussed in the previous sections, when the number of raters increases, the number of basic moves in the Markov basis grows, and the probability of selecting a non-applicable move becomes high, especially in the case of sparse tables. To overcome this problem, the definition of the stopping time *c* requires a large number of steps when the Markov basis is large and consequently the execution time increases. For large sparse tables, the algorithm needs special attention in the choice of the numerical parameters and in the optimization of the selection of the moves. A thorough study in this direction is beyond the scopes of the present paper. That is why we do not present the case of $7 \times 7 \times 7$ tables. Finally, with regard to the choice of the weights, we observe that the algorithm is a bit faster with the linear weights.

## Concluding Remarks

The analysis of the kappa-type indices through basic Markov moves presented in this paper allows us to better understand the effect of the choice of the weights and, in particular, shows that the configuration with maximum kappa strongly depends on the weights, making the normalization of the kappa statistics a non-trivial task. We have shown that, when the weights satisfy the triangular inequality, the table with maximum kappa looks quite different from that obtained with quadratic weights, and therefore, the use of distance weights should be considered as an option when choosing the weights. Since the basic moves make connected the fiber of all tables with the same margins, we have implemented a simulated annealing algorithm to actually find the configuration with maximum kappa with fixed margins in a general framework.

Future works will include the analysis of the maximum agreement when not all raters classify the same set of objects, and the speed up of the simulated annealing algorithm, especially for large sparse tables. The convergence of Markov chain-based algorithms with Markov bases for large sparse tables is a general problem in algebraic statistics, and thus, any advance in this direction would represent a notable progress also in other fields of application. Finally, we have shown that the set of all tables with a given value of weighted kappa with linear weights can be a rather large set, and it can be explored through suitable Markov bases.

## Appendix

In this appendix, the results of the simulation study with non-homogeneous margins are reported. See Sect. 5 for the description of the simulation study (Tables 3, 4).

**Table 3:** Two-rater case with non-homogeneous marginal distributions.

Weight	*k*	*N*	Mean	sd	$q_{0.99}$
Quadratic	3	20	1064.7	34.0	1172
		100	1180.8	66.7	1381
	5	20	2572.8	302.0	3599
		100	3859.4	698.4	6099
	7	20	11,265.9	1192.6	14,957
		100	15,247.9	2191.4	22,060
Linear	3	20	1039.0	24.8	1122
		100	1106.4	35.8	1205
	5	20	2294.0	208.3	3072
		100	2514.7	233.7	3283
	7	20	9989.9	863.7	13,430
		100	10,384.7	874.3	13,437
Sqrt	3	20	1058.8	33.0	1169
		100	1222.3	84.9	1457
	5	20	2528.4	274.4	3446
		100	3438.4	596.2	5350
	7	20	11,138.2	1173.5	15,031
		100	13,858.6	1982.0	19,791

Time to convergence (mean, standard deviation and 99th percentile) of the simulated annealing algorithm for different numbers of levels *k* and sample sizes *N*.

**Table 4:** Three-rater case with non-homogeneous marginal distributions.

Weight	*k*	*N*	Mean	sd	$q_{0.99}$
Quadratic	3	20	4210.5	496.6	5881
		100	5839.7	868.4	8607
	5	20	115,001.6	11,839.8	152,551
		100	145,570.5	19,149.3	210,442
Linear	3	20	4085.2	466.1	5825
		100	5395.4	775.2	7640
	5	20	110,907.2	10,893.4	146,344
		100	133,778.4	17,015.5	187,251
Sqrt	3	20	4201.5	519.9	5937
		100	5408.6	791.3	7958
	5	20	117,136.2	12,284.0	156,774
		100	136,924.9	16,926.4	187,861

Time to convergence (mean, standard deviation and 99th percentile) of the simulated annealing algorithm for different numbers of levels *k* and sample sizes *N*.
